# Supplementary material for: An IL-10/DEL-1 axis supports granulopoiesis and survival from sepsis in early life
Source: Nat Commun. 2024 Jan 23;15:680. doi: 10.1038/s41467-023-44178-y (PMC10805706; doi:10.1038/s41467-023-44178-y)
Supplement: Supplementary file 1 — Supplementary Information [file 41467_2023_44178_MOESM1_ESM.pdf]

Supplementary Materials for

An IL-10/DEL-1 axis promotes granulopoiesis and survival from sepsis in early life

Authors: Eleni Vergadi, Ourania Kolliniati, Ioanna Lapi, Eleftheria Ieronymaki, Konstantina Lyroni, Vasileia Ismini Alexaki, Eleni Diamantaki, Katerina Vaporidi, Eleftheria Hatzidaki, Helen A. Papadaki, Emmanouil Galanakis, George Hajishengallis, Triantafyllos Chavakis and Christos Tsatsanis.

The PDF file includes:

Supplementary Figure 1. The expression of ICAM-1 and CD11a in neonates.

Supplementary Figure 2. Effect of DEL-1 on monocyte population.

Supplementary Figure 3. Regulation of cytokine responses by DEL-1.

Supplementary Figure 4. Survival of WT and *Del1*$^{-/-}$ neonate mice, treated with antibiotics.

Supplementary Figure 5. The impact of DEL-1 on the neutrophil phagocytosis of non-opsonised *E. coli.*

Supplementary Figure 6. The impact of DEL-1 on bacterial clearance in the site of infection (source control).

Supplementary Figure 7. Myeloid-Derived Suppressor Cell (MDSC) population in the bone marrow of WT and DEL-1 deficient mice.

Supplementary Figure 8. SDF-1 protein expression in the bone marrow of septic WT and *Del1*$^{-/-}$ neonate mice.

Supplementary Figure 9. Expression of DEL-1 receptor CD61/CD51 in neonates.

Supplementary Figure 10. G-CSF protein expression in the serum of WT and *Del1*$^{-/-}$ neonate mice.

Supplementary Figure 11. Expression of inflammatory cytokines in the serum of WT and *Del1*$^{-/-}$ neonate mice.

Supplementary Table 1. Clinical characteristics and outcomes of the low DEL-1 and high DEL-1 groups in neonates with sepsis.

Supplementary Table 2. Clinical characteristics and outcomes in the low DEL-1 and high DEL-1 groups in adults with sepsis.

Supplementary Figure 1

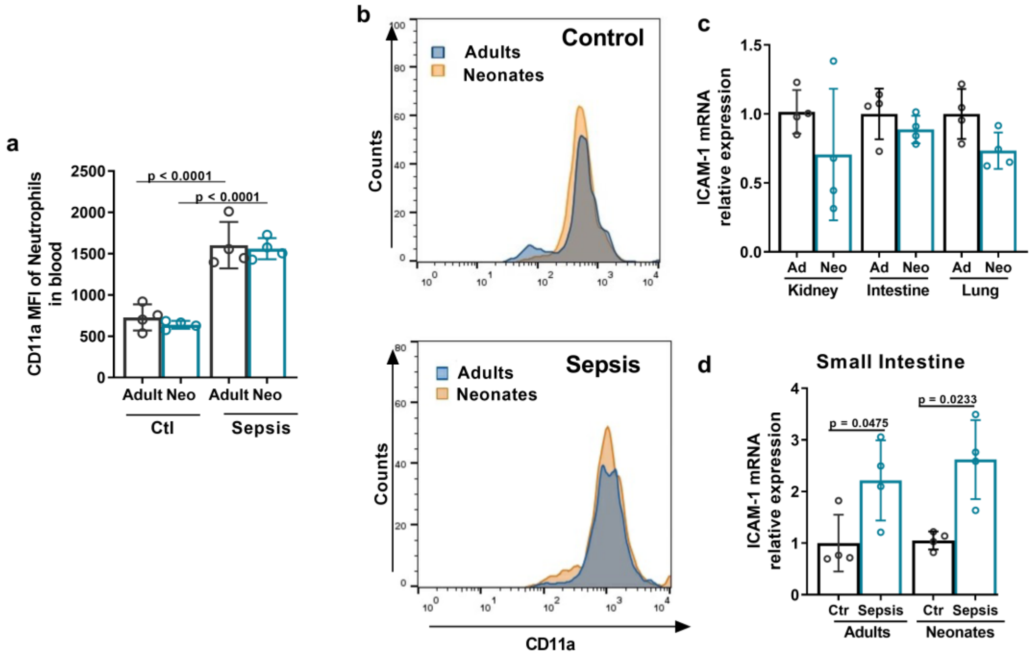

Supplementary Figure 1. **The expression of ICAM-1 and CD11a in neonates**. **a** CD11a mean fluorescence intensity (MFI) and **b** representative flow cytometry plots of (a) in neutrophils (identified as CD11b$^+$Ly6G$^+$) from control and septic adult and neonate C57BL/6 mice (n=4 animals per group). **c** ICAM-1 relative mRNA levels from kidney, intestine and lung tissue from adult and neonate C57BL/6 mice. ICAM-1 mRNA expression in each adult tissue was set as 1 (n=4 animals per group). **d** ICAM-1 relative mRNA levels from intestine of adult and neonate C57BL/6 mice after 6 hours of cecal slurry induced sepsis. ICAM-1 mRNA expression in control adult mice was set as 1 (n=4 animals per group). Statistical analysis by one-way ANOVA with Bonferroni's multiple comparison post-test (a, c) or two-sided unpaired *t* test (d). Mean ± SD is depicted (a, c, d). Source data are provided as a Source Data file. Ctl: control, Ad: adult, Neo: neonate.

Supplementary Figure 2

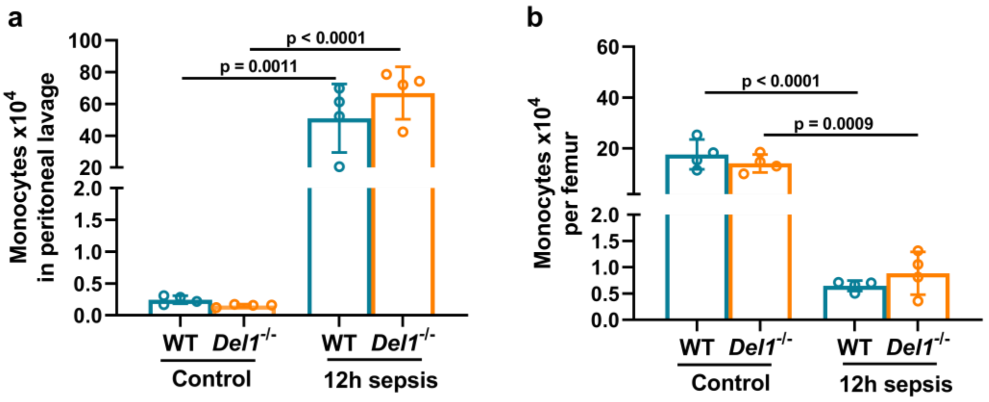

Supplementary Figure 2. **Effect of DEL-1 on monocyte population. a** Total number of monocytes (gated as $CD11b^+Ly6C^+Ly6G^-$) in peritoneal lavage (n=4 animals per group) and **b** total number of monocytes ($CD11b^+Ly6C^+Ly6G^-$) in bone marrow (n=4 animals per group) in WT and *Del1*$^{-/-}$ C57BL/6 healthy neonate mice and following 12 hours of cecal slurry - induced sepsis. Mean ± SD is depicted. Statistical analysis by one-way ANOVA with Bonferroni's multiple comparison post-test. Source data are provided as a Source Data file. h: hours.

Supplementary Figure 3

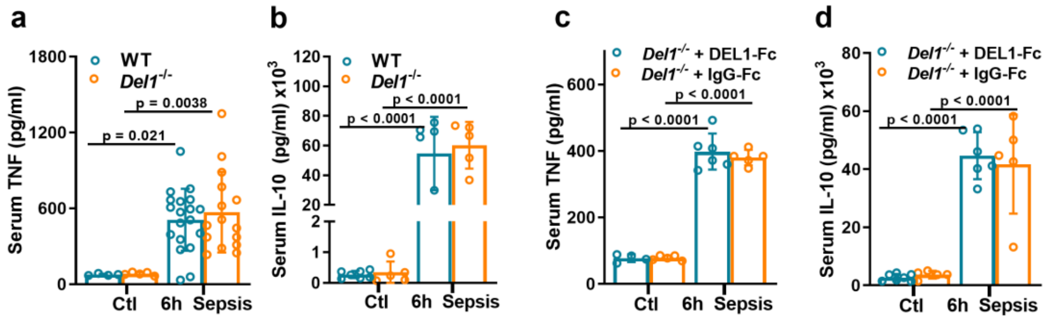

Supplementary Figure 3. **Regulation of cytokine responses by DEL-1.** Protein expression of **a** TNF (pg/ml) in serum from WT and *Del1*$^{-/-}$ C57BL/6 neonate mice in steady state and upon 6 hours of cecal slurry (CS) sepsis (n =4 animals in WT control group, n=5 animals in *Del1*$^{-/-}$ control group, n=19 animals in WT sepsis group and n=15 animals in *Del1*$^{-/-}$ sepsis group) **b** IL-10 (pg/ml) in serum from WT and *Del1*$^{-/-}$ C57BL/6 neonate mice in steady state and upon 6 hours of CS sepsis (n =6 animals in WT control group, n=5 animals in *Del1*$^{-/-}$ control group, n=6 animals in WT sepsis group and n=5 animals in *Del1*$^{-/-}$ sepsis group) **c** TNF (pg/ml) in serum from *Del1*$^{-/-}$ C57BL/6 neonate pups treated with either i.v DEL-1-Fc or IgG-Fc, in steady state and upon 6 hours after CS-induced sepsis (n=5 animals in *Del1*$^{-/-}$+IgG-Fc control group, n=4 animals in *Del1*$^{-/-}$ +DEL-1-Fc control group, n=5 animals in *Del1*$^{-/-}$ + IgG-Fc sepsis group and n=6 animals in *Del1*$^{-/-}$ +DEL-1-Fc sepsis group). **d** IL-10 (pg/ml) in serum from *Del1*$^{-/-}$ C57BL/6 neonate pups treated with either i.v DEL-1-Fc or IgG, in steady state and upon 6 hours after CS-induced sepsis (n= 5 animals in *Del1*$^{-/-}$+IgG-Fc control group, n=6 animals in *Del1*$^{-/-}$ +DEL1-Fc control group, n=5 animals in *Del1*$^{-/-}$ +IgG-Fc sepsis group and n=6 animals in *Del1*$^{-/-}$ +DEL1-Fc sepsis group). Statistical analysis by one way ANOVA with Bonferroni's multiple comparison post-test (a-d). Mean ± SD is depicted (a-d). Source data are provided as a Source Data file. Ctl: control. h: hours.

Supplementary Figure 4

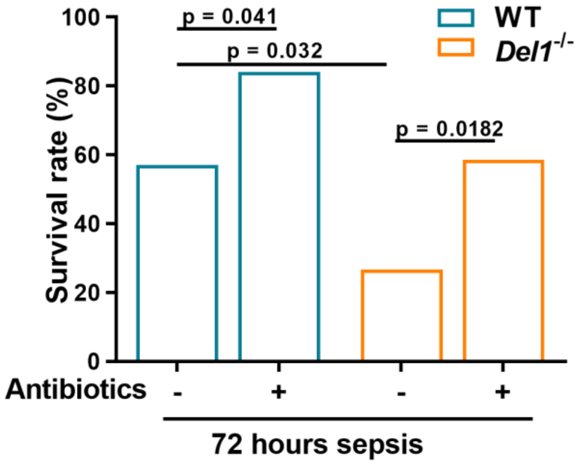

Supplementary Figure 4. Survival of WT and *Del1*$^{-/-}$ neonate mice, treated with antibiotics. Survival rate (%) of WT and *Del1*$^{-/-}$ C57BL/6 neonate mice, treated with or without antibiotics (meropenem). (n=28 animals in WT without antibiotics septic group, n=25 animals in WT with antibiotics septic group, n=30 animals in *Del1*$^{-/-}$ without antibiotics septic group and n=29 animals in *Del1*$^{-/-}$ with antibiotics septic group). Frequency % is depicted. Statistical analysis with Fisher's exact test. Source data are provided as a Source Data file.

Supplementary Figure 5

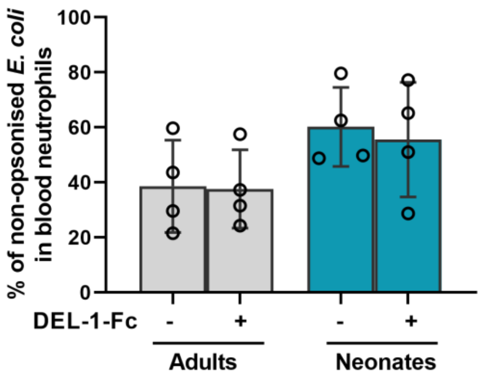

Supplementary Figure 5. The impact of DEL-1 on neutrophil phagocytosis of non-opsonised *E. coli*. Blood neutrophils ($CD11b^{+}Ly6G^{+}$) from healthy, non-septic WT adult and WT neonate mice were treated *ex vivo* with either DEL-1-Fc (10μg/ml) or same amount of IgG-Fc control and their capacity to phagocytose *ex vivo* non-opsonized FITC-labeled *E. coli* particles was assessed by flow cytometry (n=4 animals per group). Neutrophils that phagocytosed FITC-labeled *E. coli* were identified as single cells, CD11b positive, Ly6G positive and FITC - *E. coli* positive. Statistical analysis by one-way ANOVA with Bonferroni's multiple comparison post-test. Mean ± SD is depicted. Source data are provided as a Source Data file.

Supplementary Figure 6

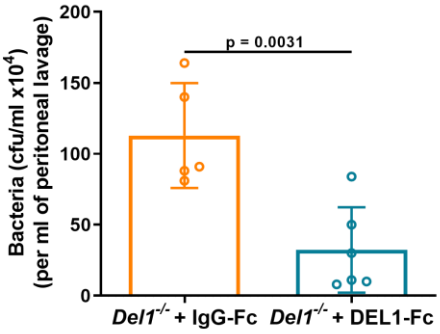

Supplemental Figure 6. The impact of DEL-1 in bacterial clearance at the site of infection (source control). Bacteria load (colony forming units, cfu) in the peritoneal lavage of septic *Del1* $^{-/-}$ C57BL/6 neonate mice (12 hours following cecal slurry induced sepsis) treated with either DEL-1-Fc (5μg/ml) or IgG-Fc control (n=5 animals in *Del1*$^{-/-}$ +IgG-Fc group and n=6 animals in *Del1*$^{-/-}$ +DEL1-Fc group). Statistical analysis by two-sided unpaired *t* test. Mean ± SD is depicted. Source data are provided as a Source Data file.

Supplementary Figure 7

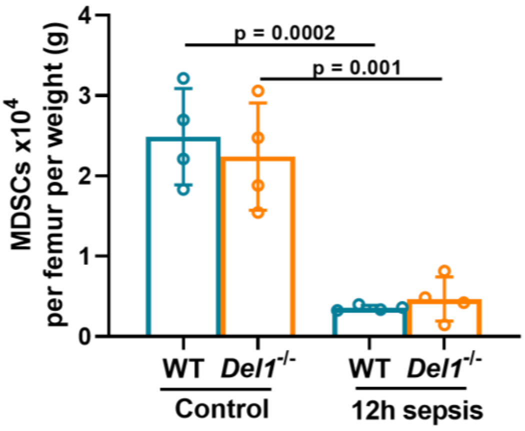

Supplementary Figure 7. Myeloid-Derived Suppressor Cell (MDSC) population in the bone marrow of WT and DEL-1 deficient mice. Total number of MDSCs (gated as $CD11c^{-}CD11b^{+}Ly6G^{+}Ly6C^{+}$) per femur per mouse weight (grams) in the bone marrow of WT and *Del1*$^{-/-}$ C57BL/6 neonate mice upon steady state and 12 hours of cecal slurry - induced polymicrobial sepsis. n= 4 mice per group. Mean ± SD is depicted. Statistical analysis by one-way ANOVA with Bonferroni's multiple comparison post-test. Source data are provided as a Source Data file. h: hours, g: grams.

Supplementary Figure 8

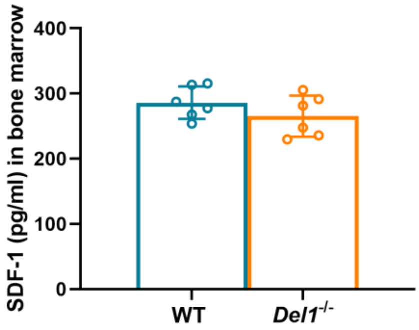

Supplementary Figure 8. SDF-1 protein expression in the bone marrow of septic WT and *Del1*$^{-/-}$ neonate mice. Protein expression of stromal cell-derived factor 1 (SDF-1) (pg/ml) in the bone marrow of WT and *Del1*$^{-/-}$ C57BL/6 neonate mice upon 12 hours of cecal slurry induced sepsis (n = 6 animals per group). Statistical analysis by two-sided unpaired *t* test. Mean ± SD is depicted. Source data are provided as a Source Data file.

Supplementary Figure 9

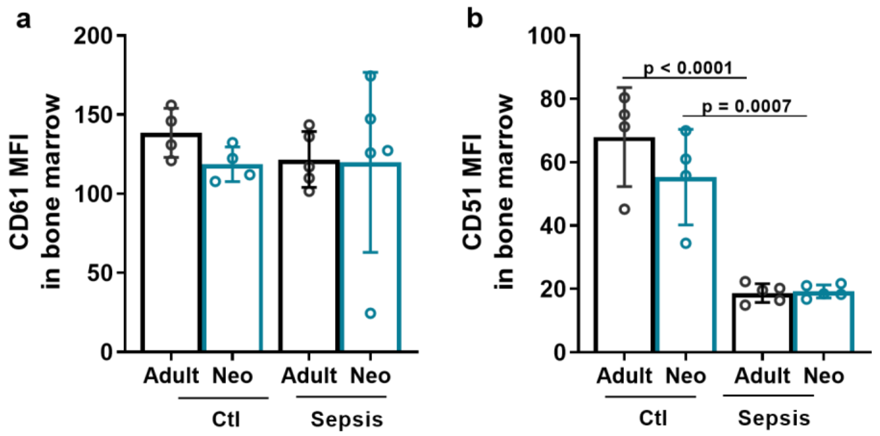

Supplementary Figure 9. **Expression of DEL-1 receptor CD61/CD51 in neonates**. **a** CD61 mean fluorescence intensity (MFI) in total bone marrow cells from adult and neonate C57BL/6 mice in steady state and upon 6 hours of cecal slurry induced sepsis (single live cells, CD61 positive, n=4 animals in adult control group, n=4 animals in neonate control group, n=5 animals in adult sepsis group and n=5 animals in adult sepsis group). **b** CD51 MFI in total bone marrow cells from adult and neonate C57BL/6 mice in steady state and upon 6 hours of cecal slurry induced sepsis (single live cells, CD51 positive, n=4 animals in adult control group, n=4 animals in neonate control group, n=5 animals in adult sepsis group and n=5 animals in adult sepsis group). Statistical analysis by one-way ANOVA with Bonferroni's multiple comparison post-test (a, b). Mean ± SD is depicted (a, b). Source data are provided as a Source Data file. Ctl: control, Neo: Neonate.

Supplementary Figure 10

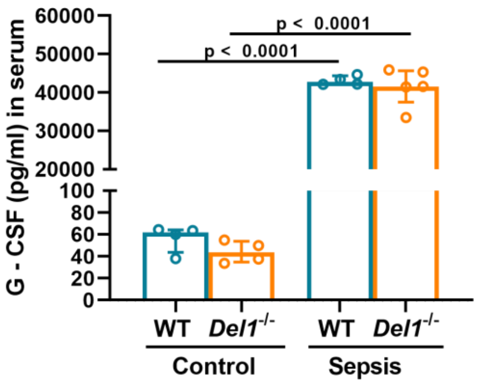

Supplementary Figure 10. G-CSF protein expression in the serum of WT and *Del1*$^{-/-}$ neonate mice. Protein expression of granulocyte colony stimulating factor (G-CSF) (pg/ml) in the serum of WT and *Del1*$^{-/-}$ C57BL/6 neonate mice upon steady state and 12 hours of cecal slurry induced sepsis (n = 4 animals in WT control group, n = 4 animals in *Del1*$^{-/-}$ control group, n = 4 animals in WT sepsis group and n = 5 animals in *Del1*$^{-/-}$ sepsis group). Statistical analysis by Kruskal-Wallis test with Dunn's multiple comparison post-test. Median ± interquartile range is depicted. Source data are provided as a Source Data file.

Supplementary Figure 11

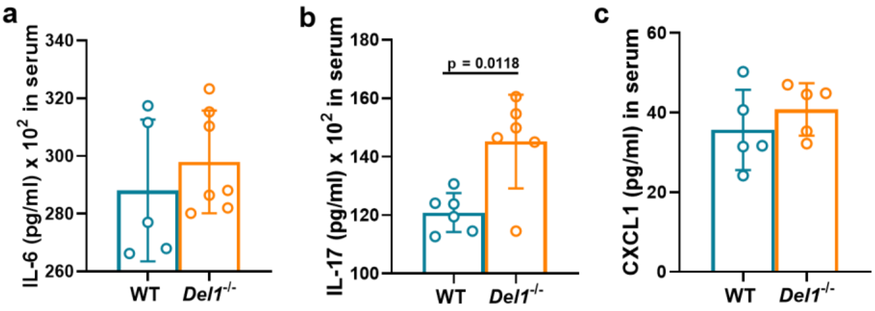

Supplementary Figure 11. Expression of inflammatory cytokines in the serum of WT and *Del1*$^{-/-}$ neonate mice upon 12 hours of sepsis. Protein expression of **a** interleukin (IL) – 6 (pg/ml) in serum (n = 5 animals in WT group and n=7 animals in *Del1*$^{-/-}$ group) **b** IL-17 (pg/ml) in serum (n = 6 animals per group) and **c** CXCL-1(pg/ml) in serum (n = 5 animals in each group), of septic WT and *Del1*$^{-/-}$ C57BL/6 neonate mice (12 hours of cecal slurry sepsis). Statistical analysis by two-sided unpaired *t* test (a, b, c). Mean ± SD is depicted (a, b, c). Source data are provided as a Source Data file.

Supplementary Table 1. Clinical characteristics and outcomes of the low DEL-1 and high DEL-1 groups in neonates with sepsis.

	All cases n = 24	Low DEL-1 n = 12	High DEL-1 n = 12	p value*
**Demographics**				
Sex (males)	15/24 (62.5%)	8/12 (66.7)	7/12 (58.3%)	>0.9999
Postnatal days†	2 (1 -4)	1.5 (1 – 3)	2.5 (1 – 4)	0.8877
Gestational age ǂ (weeks)ǂ	34.36 (±3.6)	34.37 (±2.9)	34.35 (±3.4)	0.9848
**Type of sepsis**				
Early onset	20/24 (83.3%)	10/12 (83.3%)	10/12 (83.3%)	>0.9999
Late onset	4/24 (16.6%)	2/12 (16.6%)	2/12 (16.6%)	>0.9999
Bacteremia	7/24 (29.2%)	4/12 (33.3%)	3/12 (25%)	>0.9999
**Laboratory data**				
IL-6 (pg/ml) †	188.7 (87-391)	186.2 (62-338)	188.7 (101-2578)	0.10774
IL-10 to IL-17 ratio ǂ	7.26 (20.03)	0.32 (±0.32)	14.20 (±20.07)	**0.0355**
ANC (x10^3/μl) ǂ Day 0	6,040 (±3.390)	6,880 (± 4,320)	5,190 (±3,890)	0.3269
ANC (x10^3/μl)† Day 1	6,003(3,525-8,520)	5,280 (2,076-8,266)	6,444 (3,685-13,250)	0.5563
ANC (fold increase) †	1.62 (±1.3)	1 (±0.4)	2.3 (±1.28)	**0.00717**
**Outcome**				
ICU length of stay†	14 (10-30.7)	12 (10-30.50)	14 (10-31)	0.8051
28 – day mortality	4/24 (16.7%)	3/12 (25%)	1/12 (8.33%)	0.5901

Abbreviations: Low DEL-1 group: serum DEL-1 levels less than 700pg/ml; ANC: absolute neutrophil count, ICU: intensive care unit.

Definitions: early onset: sepsis in neonates less than 7 days old; late-onset: sepsis in neonates≥ 7 days old; *Comparison among low and high DEL-1 group; statistical analysis was performed with Fisher's exact test or Chi-squared test (for categorical data) and two-sided unpaired *t* test (ǂ) or by two-sided Mann-Whitney test (†) for numerical data. ǂMean ±SD and †median (interquartile range) are depicted. In ANC (fold increase) the Low DEL-1 group value was set to 1. Source data are provided as a Source Data file. The DEL-1 protein level data, the ANC on day 0 and day and the mortality rate data presented in this table are also depicted in Figure 5 and figure 9h of the main manuscript.

Supplementary Table 2. Clinical characteristics and outcomes in the low DEL-1 and high DEL-1 groups in adults with sepsis

	All cases<br>n = 40	Low DEL-1<br>n = 25	High DEL-1<br>n = 15	p value*
**Demographics**				
Sex (males)	27/40 (67.5%)	17/25 (68%)	10/15 (66.7%)	>0.9999
Age in years†	66 (46 - 72)	68 (47 - 75)	54 (41 - 68)	0.1071
SOFA score (median, range)	8 (6-10)	8 (6-10)	9 (6.5-12.5)	0.5025
**Source of sepsis**				
Pulmonary	24/40 (60%)	13/25 (52%)	11/15 (73.3%)	0.3176
Abdominal	10/40 (25%)	8/25 (20%)	2/15 (13.3%)	0.2686
Other	6/40 (15%)	4/25 (26.5%)	2/15 (13.3%)	>0.9999
Positive Blood cultures	13/40 (32.5%)	8/25 (20%)	5/15 (33.3%)	>0.9999
**Laboratory data**				
IL-6 (pg/ml) †	410 (179 – 1400)	403 (161 – 1834)	378 (193–1093)	0.6422
**Outcome**				
ICU length of stay †	21 (13-29)	21 (13-31)	17 (12-30)	0.6221
28 – day mortality	13/40 (32.5%)	11/25 (44%)	2/15 (13.3%)	**0.0450#**

Abbreviations: Low DEL-1 Group: serum DEL-1 levels less than 125pg/ml, ANC: absolute neutrophil count. ICU: intensive care unit.

*Comparison among low and high DEL-1 group; statistical analysis was performed with Fisher's exact test or Chi-squared test (#) (for categorical data) and by two-sided Mann-Whitney test (†) for numerical data. † Median (interquartile range) is depicted. Source data are provided as a Source Data file. The DEL-1 protein level data and the mortality rate data presented in this table are also depicted in Figure 5 of the main manuscript.
