# Supplementary material for: An IL-10/DEL-1 axis supports granulopoiesis and survival from sepsis in early life
Source: Nat Commun. 2024 Jan 23;15:680. doi: 10.1038/s41467-023-44178-y (PMC10805706; doi:10.1038/s41467-023-44178-y)
Supplement: Supplementary file 3 — Reporting Summary [file 41467_2023_44178_MOESM3_ESM.pdf]

nature portfolio

Corresponding author(s): Eleni Vergadi

Last updated by author(s): Nov 4, 2023

Reporting Summary

Nature Portfolio wishes to improve the reproducibility of the work that we publish. This form provides structure for consistency and transparency in reporting. For further information on Nature Portfolio policies, see our Editorial Policies and the Editorial Policy Checklist.

Statistics

For all statistical analyses, confirm that the following items are present in the figure legend, table legend, main text, or Methods section.

n/a	Confirmed	
☐	☒	The exact sample size (*n*) for each experimental group/condition, given as a discrete number and unit of measurement
☐	☒	A statement on whether measurements were taken from distinct samples or whether the same sample was measured repeatedly
☐	☒	The statistical test(s) used AND whether they are one- or two-sided<br>*Only common tests should be described solely by name; describe more complex techniques in the Methods section.*
☐	☒	A description of all covariates tested
☐	☒	A description of any assumptions or corrections, such as tests of normality and adjustment for multiple comparisons
☐	☒	A full description of the statistical parameters including central tendency (e.g. means) or other basic estimates (e.g. regression coefficient) AND variation (e.g. standard deviation) or associated estimates of uncertainty (e.g. confidence intervals)
☐	☒	For null hypothesis testing, the test statistic (e.g. *F*, *t*, *r*) with confidence intervals, effect sizes, degrees of freedom and *P* value noted<br>*Give P values as exact values whenever suitable.*
☒	☐	For Bayesian analysis, information on the choice of priors and Markov chain Monte Carlo settings
☒	☐	For hierarchical and complex designs, identification of the appropriate level for tests and full reporting of outcomes
☒	☐	Estimates of effect sizes (e.g. Cohen's *d*, Pearson's *r*), indicating how they were calculated

Our web collection on statistics for biologists contains articles on many of the points above.

Software and code

Policy information about availability of computer code

Data collection	N/A
Data analysis	N/A

For manuscripts utilizing custom algorithms or software that are central to the research but not yet described in published literature, software must be made available to editors and reviewers. We strongly encourage code deposition in a community repository (e.g. GitHub). See the Nature Portfolio guidelines for submitting code & software for further information.

Data

Policy information about availability of data

All manuscripts must include a data availability statement. This statement should provide the following information, where applicable:

- Accession codes, unique identifiers, or web links for publicly available datasets
- A description of any restrictions on data availability
- For clinical datasets or third party data, please ensure that the statement adheres to our policy

No high throughput data are generated within this work. No restrictions apply for data availability. All data are provided in an supplementary Source data file.

Human research participants

Policy information about studies involving human research participants and Sex and Gender in Research.

Reporting on sex and gender	For animals and humans included in this study both sexes were included. No sex/gender specific analysis was carried out.
Population characteristics	yes
Recruitment	yes
Ethics oversight	Institutional Review Board of the University General Hospital of Heraklion (the approval number protocols are mentioned in the manuscript).

Note that full information on the approval of the study protocol must also be provided in the manuscript.

Field-specific reporting

Please select the one below that is the best fit for your research. If you are not sure, read the appropriate sections before making your selection.

☒ Life sciences ☐ Behavioural & social sciences ☐ Ecological, evolutionary & environmental sciences

For a reference copy of the document with all sections, see nature.com/documents/nr-reporting-summary-flat.pdf

Life sciences study design

All studies must disclose on these points even when the disclosure is negative.

Sample size	Sample size for the in vivo experiments in mouse models was calculated according to G*Power analysis and is part of the license obtained for performing these experiments.
Data exclusions	No data have been excluded in the analyses
Replication	Replication of the experiments is noted in the Materials and Methods and figure legends, where the number of biological replicates and/or experiments performed is indicated.
Randomization	N/A
Blinding	Blinding was not possible due to the nature of the experiments.

Behavioural & social sciences study design

All studies must disclose on these points even when the disclosure is negative.

Study description	N/A
Research sample	N/A
Sampling strategy	N/A
Data collection	N/A
Timing	N/A
Data exclusions	N/A
Non-participation	N/A
Randomization	N/A

Ecological, evolutionary & environmental sciences study design

All studies must disclose on these points even when the disclosure is negative.

Study description	N/A
Research sample	N/A
Sampling strategy	N/A
Data collection	N/A
Timing and spatial scale	N/A
Data exclusions	N/A
Reproducibility	N/A
Randomization	N/A
Blinding	N/A

Did the study involve field work? ☐ Yes ☒ No

Reporting for specific materials, systems and methods

We require information from authors about some types of materials, experimental systems and methods used in many studies. Here, indicate whether each material, system or method listed is relevant to your study. If you are not sure if a list item applies to your research, read the appropriate section before selecting a response.

Materials & experimental systems

n/a	Involved in the study	
☐	☒	Antibodies
☐	☒	Eukaryotic cell lines
☒	☐	Palaeontology and archaeology
☐	☒	Animals and other organisms
☐	☒	Clinical data
☒	☐	Dual use research of concern

Methods

n/a	Involved in the study	
☒	☐	ChIP-seq
☐	☒	Flow cytometry
☒	☐	MRI-based neuroimaging

Antibodies

Antibodies used

Anti-Mouse CD16/CD32 (Mouse Fc Block) (1:200, catalog no. 553141, BD Pharmigen, San Diego, CA, USA)
PE anti-mouse Ly6G (1:200, 1A8-Ly6g, catalog no. 12-9668-82, E-bioscience, San Diego, CA, USA)
PE- anti- mouse Ly6G (1:200, 1A8, catalog no. 127608, Biolegend, San Diego, CA), APC anti-mouse CD11b (1:100, M1/70, catalog no. 101229, Biolegend, San Diego, CA, USA)
FITC anti-mouse CD11b antibody (1:100, M1/70, catalog no. 101205, Biolegend, San Diego, CA, USA)
PE anti-mouse Ly6G (1:200, 1A8-Ly6g, catalog no. 12-9668-82, E-bioscience, San Diego, CA, USA)
PE- anti- mouse Ly6G (1:200, 1A8, catalog no. 127608, Biolegend, San Diego, CA)
APC anti-mouse Ly6C (1:200, HK1.4, catalog no. 128016, Biolegend, San Diego, CA, USA)
PerCP/Cy5.5 anti-mouse CD11c (1:200, N418, catalog no. 117328, Biolegend, San Diego, CA, USA)
PE anti-mouse CD51 (1:200, RMV-7, catalog no. 104105, Biolegend, San Diego, CA, USA)
FITC anti-mouse CD61 (1:100, catalog no. 10430, Biolegend, San Diego, CA, USA)
PE-Cy7 anti-mouse Sca-1 antibody (1:100, D7, catalog no. 108113, Biolegend, San Diego, CA, USA)
PE anti-mouse CD34 (1:50, MEC14.7, catalog no. 119307, Biolegend, San Diego, CA, USA)
APC anti-mouse Lineage antibody cocktail (1:10, catalog no. 558074, BD Pharmigen, San Diego, CA, USA)
BV480 anti-mouse CD117 (c-Kit) (1:100, 2B8, catalog no. 566074, BD Pharmigen San Diego, CA, USA)
BV421 anti-mouse CD16/32 (1:50, catalog no. 101331, Biolegend, San Diego, CA, USA)
Alexa Fluor 700 anti-mouse CD48 (1:50, HM48-1, catalog no. 103425, Biolegend, San Diego, CA)
PerCP/Cy5.5 anti-mouse CD150 (SLAM) (1:50, TC15-12F12.2, catalog no. 115921, Biolegend, San Diego, CA)
PE- anti-mouse CD135 (Ftl3) (1:50, A2F10, catalog no. 135305, Biolegend, San Diego, CA)
Recombinant IgG1 Fc protein carrier free (catalog no. 110-HG, R&D Systems, Minneapolis, USA)
Purified endotoxin-free anti-mouse IL-10R blocking antibody (Purified anti-mouse CD210 antibody, low endotoxin, azide-free, clone 1B1.3a, catalog no. 112710, Biolegend, San Diego, CA, USA)

	Purified Rat IgG1, κ Isotype Ctrl Antibody, low endotoxin, azide-free, clone RTK2071, catalog no. 400432, Biolegend, San Diego, CA, USA)
Validation	Validation details for each primary antibody can be found in manufacturer website

Eukaryotic cell lines

Policy information about cell lines and Sex and Gender in Research

Cell line source(s)	HUVEC cells were purchased from Lonza (Basel, Switzerland).<br>Ea.hy926 endothelial cell line were kindly provided by Prof. Kardasis, University of Crete, School of Medicine.<br>Primary bone marrow cells and blood neutrophils were isolated from mice of neonatal and adult age. Both males and female mice were used, but sex was not tracked as a biological as not differences were noted in DEL-1 expression. Mesenchymal Stromal Cells (MSCs) were isolated from Wharton jelly (after informed consent). Isolation and culture of Mesenchymal Stromal Cells (MSCs) have been approved by Ethics Committee of the University Hospital of Heraklion, Crete, Greece (approval number 1724).
Authentication	Yes.
Mycoplasma contamination	All cell lines tested negative for mycoplasma contamination.
Commonly misidentified lines<br>(See ICLAC register)	The cell lines used in this study were not identified in the list of known misidentified cell lines maintained by the International Cell Line Authentication Committee .

Palaeontology and Archaeology

Specimen provenance	N/A
Specimen deposition	N/A
Dating methods	N/A

☐ Tick this box to confirm that the raw and calibrated dates are available in the paper or in Supplementary Information.

Ethics oversight	N/A

Note that full information on the approval of the study protocol must also be provided in the manuscript.

Animals and other research organisms

Policy information about studies involving animals; ARRIVE guidelines recommended for reporting animal research, and Sex and Gender in Research

Laboratory animals	Mice of C57BL/6 background, either WT of DEL-1 deficient (Edil3-/-) were used.
Wild animals	C57BL/6 wild type mice of adult (8-10weeks old) and neonatal age were used.
Reporting on sex	Both male and female mice and humans were used for experiments. Sex was not tracked as a biological variable, as no difference related to DEL-1 expression or immune function, particularly on neonatal age were observed between the male and female.
Field-collected samples	N/A
Ethics oversight	All animal experimentation was in adherence to the “NIH Guide for the Care and Use of Laboratory Animals” and all animal procedures were in accordance with institutional guidelines and were approved by the University of Crete’s Animal Care and Use Committee and the Veterinary Department of the Heraklion Prefecture (license number 150760 and 6540). For all analyses on human samples, informed, written consent was obtained from all participants at the time of the recruitment, and the studies were conducted in accordance with the Helsinki Declaration ethical standards. No compensation was provided to participants included in this study. All procedures were conducted upon approval of the Institutional Review Board of the University General Hospital of Heraklion (approval numbers 1724, 2418 and 375047). Isolation and culture of Mesenchymal Stromal Cells (MSCs) was performed after informed consent and has been approved by Ethics Committee of the University Hospital of Heraklion, Crete, Greece (approval number 1724).

Note that full information on the approval of the study protocol must also be provided in the manuscript.

Clinical data

Policy information about clinical studies

All manuscripts should comply with the ICMJE guidelines for publication of clinical research and a completed CONSORT checklist must be included with all submissions.

Clinical trial registration	N/A
Study protocol	N/A
Data collection	N/A
Outcomes	N/A

Dual use research of concern

Policy information about dual use research of concern

Hazards

Could the accidental, deliberate or reckless misuse of agents or technologies generated in the work, or the application of information presented in the manuscript, pose a threat to:

No	Yes	
☒	☐	Public health
☒	☐	National security
☒	☐	Crops and/or livestock
☒	☐	Ecosystems
☒	☐	Any other significant area

Experiments of concern

Does the work involve any of these experiments of concern:

No	Yes	
☒	☐	Demonstrate how to render a vaccine ineffective
☒	☐	Confer resistance to therapeutically useful antibiotics or antiviral agents
☒	☐	Enhance the virulence of a pathogen or render a nonpathogen virulent
☒	☐	Increase transmissibility of a pathogen
☒	☐	Alter the host range of a pathogen
☒	☐	Enable evasion of diagnostic/detection modalities
☒	☐	Enable the weaponization of a biological agent or toxin
☒	☐	Any other potentially harmful combination of experiments and agents

ChIP-seq

Data deposition

☐ Confirm that both raw and final processed data have been deposited in a public database such as GEO.

☐ Confirm that you have deposited or provided access to graph files (e.g. BED files) for the called peaks.

Data access links<br>*May remain private before publication.*	N/A
Files in database submission	N/A
Genome browser session<br>(e.g. UCSC)	N/A

Methodology

Replicates	N/A
Sequencing depth	N/A

Antibodies	N/A
Peak calling parameters	N/A
Data quality	N/A
Software	N/A

Flow Cytometry

Plots

Confirm that:

☒ The axis labels state the marker and fluorochrome used (e.g. CD4-FITC).

☒ The axis scales are clearly visible. Include numbers along axes only for bottom left plot of group (a 'group' is an analysis of identical markers).

☒ All plots are contour plots with outliers or pseudocolor plots.

☒ A numerical value for number of cells or percentage (with statistics) is provided.

Methodology

Sample preparation	Total white blood cell (WBC) counts of peritoneal lavage isolated cells, whole blood and bone marrow cells were isolated from mice, assessed by hemocytometer counting using acetic acid 3% treatment, and were placed in flow cytometry staining buffer. For GMP and bone marrow progenitor cell analysis, primary bone marrow total cells were collected from mice. Erythrocytes were removed via incubation with ACK lysing buffer (Thermofisher, Invitrogen, Carlsbad, CA, USA). Cell suspension was then counted as described above and was kept frozen in FBS supplemented with 10% DMSO at -80oC and then in liquid nitrogen for up to 1 month prior to use.
Instrument	FACS Canto II (BD Biosciences, San Jose, CA)
Software	FlowJo v10.7.1 Software (BD Biosciences, San Jose, CA)
Cell population abundance	Cell sorting was not performed
Gating strategy	Blood neutrophils were identified as single cells, CD11b positive, Ly6G positive and Ly6C negative. Monocytes were identified as single cells, CD11b positive, Ly6G negative and Ly6C positive. Neutrophils that phagocytosed FITC-labeled E. coli were identified as single cells, CD11b, Ly6G positive and FITC- E. coli positive. MDSCs were identified as single cells, CD11c negative, CD11b positive, and Ly6G and Ly6C positive. GMP progenitor cells were identified as single cells, Lineage neg, CD117 (c-Kit) positive, Sca-1 negative and CD16/32 and CD34 positive. Gating strategies for HSPC were as follows: LSK, Lin −Sca-1+cKit+; LT-HSC, CD48−CD150+LSK; ST-HSC, CD48−CD150−LSK; MPP, CD48+CD150−LSK; MPP2, Flt3−CD48+CD150+LSK; MPP3, Flt3−CD48+CD150−LSK.

☒ Tick this box to confirm that a figure exemplifying the gating strategy is provided in the Supplementary Information.

Magnetic resonance imaging

Experimental design

Design type	N/A
Design specifications	N/A
Behavioral performance measures	N/A

Acquisition

Imaging type(s)	N/A
Field strength	N/A
Sequence & imaging parameters	N/A
Area of acquisition	N/A

Diffusion MRI ☐ Used ☒ Not used

Preprocessing

Preprocessing software	N/A
Normalization	N/A
Normalization template	N/A
Noise and artifact removal	N/A
Volume censoring	N/A

Statistical modeling & inference

Model type and settings	N/A
Effect(s) tested	N/A

Specify type of analysis: ☐ Whole brain ☐ ROI-based ☐ Both

Statistic type for inference (See Eklund et al. 2016)	N/A
Correction	N/A

Models & analysis

n/a	Involved in the study
☒	☐ Functional and/or effective connectivity
☒	☐ Graph analysis
☒	☐ Multivariate modeling or predictive analysis
